# Existing and Emerging Approaches to Risk Assessment in Patients with Ascending Thoracic Aortic Dilatation

**DOI:** 10.3390/jimaging8100280

**Published:** 2022-10-14

**Authors:** Nina D. Anfinogenova, Valentin E. Sinitsyn, Boris N. Kozlov, Dmitry S. Panfilov, Sergey V. Popov, Alexander V. Vrublevsky, Alexander Chernyavsky, Tatyana Bergen, Valery V. Khovrin, Wladimir Yu. Ussov

**Affiliations:** 1 Cardiology Research Institute, Tomsk National Research Medical Center, Russian Academy of Sciences, Tomsk 634012, Russia; 2 University Hospital, Lomonosov Moscow State University, Moscow 119991, Russia; 3 E. Meshalkin National Medical Research Center, Novosibirsk 630055, Russia; 4 Petrovsky National Research Centre of Surgery, Moscow 119991, Russia

**Keywords:** ascending thoracic aortic aneurysm, risk prediction, open aortic repair, imaging, biomechanics, pre-emptive surgery, transdisciplinary

## Abstract

Ascending thoracic aortic aneurysm is a life-threatening disease, which is difficult to detect prior to the occurrence of a catastrophe. Epidemiology patterns of ascending thoracic aortic dilations/aneurysms remain understudied, whereas the risk assessment of it may be improved. The electronic databases PubMed/Medline 1966–2022, Web of Science 1975–2022, Scopus 1975–2022, and RSCI 1994–2022 were searched. The current guidelines recommend a purely aortic diameter-based assessment of the thoracic aortic aneurysm risk, but over 80% of the ascending aorta dissections occur at a size that is lower than the recommended threshold of 55 mm. Moreover, a 55 mm diameter criterion could exclude a vast majority (up to 99%) of the patients from preventive surgery. The authors review several visualization-based and alternative approaches which are proposed to better predict the risk of dissection in patients with borderline dilated thoracic aorta. The imaging-based assessments of the biomechanical aortic properties, the Young’s elastic modulus, the Windkessel function, compliance, distensibility, wall shear stress, pulse wave velocity, and some other parameters have been proposed to improve the risk assessment in patients with ascending thoracic aortic aneurysm. While the authors do not argue for shifting the diameter threshold to the left, they emphasize the need for more personalized solutions that integrate the imaging data with the patient’s genotypes and phenotypes in this heterogeneous pathology.

## 1. Introduction

Ascending thoracic aortic aneurysm is a life-threatening and insidious disease that is difficult to detect prior to the occurrence of a catastrophe [1,2]. A single-center study, which was based on computed tomography (CT) scans that included the chest, which were performed in the Yale University School of Medicine, allowed for the authors to evaluate the prevalence of the incidentally detected ascending thoracic aortic dilations with the ascending thoracic aorta diameter ≥4.0 cm: 2.1% of the total, and 3.2% and 0.9% in men and women, respectively, and 2.8% in people who were aged ≥50 years [3]. A population-based retrospective cohort study, which was carried out in Ontario, Canada, showed that the incidence rate of type A aortic dissections (TAD) is 1.5 per 100,000, and total rate of the incidentally detected thoracic aortic aneurysms is 7.6 per 100,000 [4]. However, the true incidence of ascending thoracic aortic dilations including the undetected cases in the population remains poorly studied.

The timely diagnosis and pre-emptive management of it are essential, and patients may benefit from an elective aortic repair if a twelve-month risk of the aneurysm rupture/dissection exceeds the combined risks of the perioperative morbidity and mortality. The patients with thoracic aortic aneurysms who receive later elective surgeries experience significantly worse outcomes when they are compared with the medically triaged candidates [5]. The current guidelines suggest that an aortic diameter-based assessment of the thoracic aortic aneurysm risk should be conducted [6] and recommend pre-emptive open aortic repair if the aortic diameter reaches 55 mm because greater diameters are associated with a significantly higher risk of dissection or rupture. However, most of the ascending aorta dissections occur at a size that is lower than the recommended threshold.

The questions on the personalization of the indications for the pre-emptive open repair and potential left-shift of diameter-based criterion continue to be discussed in the literature. Moreover, alternative approaches to assessing the risk of ascending aortic aneurysm dissection/rupture are currently under development, and they may potentially result in the elaboration of more precise tools to select the patients for pre-emptive open aortic repair. A personalized risk assessment may be the most promising strategy for establishing adequate indications for the pre-emptive open repair of ascending thoracic aorta. The aim of this article is to discuss the approaches to a personalized risk prediction in ascending thoracic aortic disease.

To find the relevant literature data, the electronic databases such as PubMed/Medline (1966–2022), Web of Science (1975–2022), Scopus (1975–2022), and Russian Science Citation Index (RSCI) (1994–2022) were searched. The search terms were as follows: ascending aorta, thoracic aorta, aortic dilatation, thoracic aortic aneurism, aortic aneurysm, aortic dissection, computed tomography, echocardiography, magnetic resonance imaging, ultrasound, hemodynamics, machine learning, prophylactic surgery, prediction, and risk. The Web of Science Core Collection, Scopus, and RSCI data were used to find, analyze, and review the citation reports and papers that cited the relevant publications, which were retrieved during the primary search. After an initial retrieval of 3615 results, 70 papers were selected based on the criteria of relevance, novelty, and for the avoidance of duplication.

## 2. Assessment of Aortic Dimensions for Risk Stratification

### 2.1. Aortic Diameter

The current guidelines recommend that a pre-emptive open aortic repair be conducted if the aortic diameter reaches 55 mm. However, a study, which is based on serial imaging examinations, suggested that the aortic diameter of 55 mm may be an imperfect predictor of an aortic catastrophe [6]. The study found that the mean ascending aorta sizes were 54.2 ± 7.0 and 45.1 ± 5.7 mm at dissection and prior to it, respectively, whereas the dissection itself significantly increased the aortic size by 7.65 mm. The study by Mansour et al. demonstrated that over 80% of the ascending aorta dissections might occur at a size that is lower than the recommended threshold of 55 mm [7]. Yet, another study showed that a 55 mm diameter criterion could exclude ∼99% of patients from the preventive surgery [8]. Ziganshin et al. generated the data encouraging the shift of an aortic diameter-based criterion to the left in some patients, and the authors proposed to implement the “left-shift” to 50 mm in large, specialized centers or by surgeons who show an excellent performance of open ascending aortic repair [9]. At the same time, the study of Monaghan et al. supported the current guidelines and demonstrated the acceptable survival outcomes in asymptomatic bicuspid aortic valve (BAV) patients without a family history of it and with a proximal aortic diameter of 50–55 mm. The patients were monitored using CT techniques or transesophageal echocardiography (TEE) until they became symptomatic, or the aortic diameter exceeded 55 mm. The outcomes in these patients were similar to those who were immediately operated on upon the detection of a proximal aortic diameter that was within 50–55 mm. This study, however, also has some limitations as it is a single-center retrospective research study with a relatively small sample size while the groups differ in their confounding variables [10].

### 2.2. Aortic Size Indexes

Zafar et al. showed that indexing the absolute aortic size to the biometric data is a valid tool for a risk estimation of the occurrence of rupture, dissection, or death in patients with ascending thoracic aortic aneurysms. It allows the authors to compensate for the risk differences that are skewed by stature, though the aortic sizes and behaviors are significantly influenced by sex [11]. This predictive tool that is based on height only improved the prediction power, and the epidemiological analysis retrospectively showed that a height-only prediction tool potentially protected 91% of patients from a thoracic aortic ascending aneurysm dissection [11]. Patients with small body sizes require a prompter surgery because they have a higher aortic-to-body-size ratio which indicates an increased risk. The aortic size index, a ratio of aortic diameter (cm) to body surface area (cm^2^), correlates with aortic catastrophes and deaths, and an index of 4.25 cm/m^2^ is associated with 20% annual risk [12]. The aortic size index also correlates with maximal rates of systolic distension and diastolic recoil, which may be markers of the aortic stiffness increasing. An analysis of the relationship between the aortic size index, the maximal rate of systolic distension, and the maximal rate of diastolic recoil characterizing the severity of the disease allowed the authors to compensate for having a small study population and no healthy, matched control group [13].

### 2.3. Aortic Length

The aortic length characterizes the natural history of the ascending thoracic aortic aneurysm, and the measure of it is used to develop the new predictive tools to improve the risk stratification [14,15,16,17]. It is measured as the distance from the aortic annulus to the origin of the innominate artery [14]. Wu et al. introduced the aortic height index, which is calculated as diameter height index + length height index. The authors showed that the aortic height index including both the length and diameter is easily discernible via modern imaging modalities, and it is more powerful than the diameter is alone in predicting the long-term aortic adverse events, with it having an increased area under the curve. The comprehensive statistical analysis showed that a 110 mm aortic elongation may serve as an intervention criterion for ascending thoracic aortic aneurysm [14]. Heuts et al. also showed that the assessments of the aortic volume and length are superior in their diagnostic accuracy when they are compared with that of the maximum diameter. These authors expect that lowering the threshold to 50 mm would increase diameter sensitivity from 4% to 17%. The sensitivities of the aortic volume (from 20% to 38%) and length (from 28% to 33%) may also increase. These studies are also limited by them having a cross-sectional design and a relatively small study population [15]. The presence of ascending aorta dilation along with the elongation of it enabled Krüger et al. to identify significantly more pre-TAD patients when it was compared with only using diameter alone. The patients with dilated (45–54 mm) and elongated (≥120 mm) ascending aortas represent high-risk candidates for pre-emptive surgery though this study is also retrospective, and the group of patients that was used is small [16]. Accurate aortic length measurements also allow for precise pulse wave velocity assessments, which are helpful for the risk stratification [17].

### 2.4. Measurements of Aortic Dimensions

Magnetic resonance imaging (MRI) provides accurate dimensional measurements of the aortic root/ascending aorta/aortic arch. It provides an excellent image quality in the motion-prone segments with a short acquisition time [18,19]. Besides the aortic dimensions, the MRI also allows for the direct and precise characterization of the curvature, the aortic wall thickness, and the functional parameters including the aortic strain, its distensibility, and the pulse wave velocity. The superb reproducibility of the MRI methods allows practitioners to monitor the responses to the treatments. Three-dimensional phase-contrast velocity 4D flow MRI can further characterize the altered aortic geometry and function [20]. The geometry of the ventriculo-aortic junction and the blood flow patterns are shown to be essential for the progression of the ascending thoracic aortic aneurysm disease. The MRI-based left ventricular outflow tract aortic angle may be a predictor of the disease severity [21]. The measurements of the ascending aorta through the use of contrast-enhanced CT angiographies are comparable with those that are acquired through the use of contrast- and radiation-free self-navigated 3D whole-heart magnetic resonance angiography [19]. Transthoracic echocardiography (TTE) provides accurate measurements of the aortic root diameter [22], but the imaging of the aortic arch as well as mid and distal ascending aorta is challenging, whereas the echocardiogram inter- and intra-observation differences reach high values. The TEE method allows researchers to image the aortic root, the ascending aorta, and the aortic arch, and it may be used when both CT scans and MRIs are contraindicated. However, the TEE method requires sufficient sedation and blood pressure control during the study [23].

## 3. Central Hemodynamic Measurements

A proof-of-concept study identified three hemodynamic measures that were superior to the current standard of care (aneurysm size) in the evaluation of future aneurysm growth. The measures included the carotid-femoral pulse wave velocity, the central pulse pressure, and the forward pressure wave amplitude. Among these, the carotid-femoral pulse wave velocity and the central pulse pressure require only the arterial tonometry for their assessment, and they are proposed for future simple and easily adoptable clinical algorithms to predict the thoracic aortic aneurysm expansion [24]. Other studies have highlighted a significance of the adverse pulsatile hemodynamics in the thoracic aortic aneurysms in women, and they encourage the use of sex-specific strategies during the risk assessment of them, the monitoring of them, and the treatment to improve the thoracic aortic aneurysm-related outcomes. In sex-specific models, a higher carotid-femoral pulse wave velocity, a global reflection coefficient, and a central systolic blood pressure are associated with a larger aneurysm size in women, but not in men. The carotid-femoral pulse wave velocity and central systolic blood pressure are significantly associated with larger aneurysms in both of the sexes [25]. However, the findings in these studies seem to be limited due to the administration of different imaging modalities and them having a small sample size.

## 4. Biomechanical Properties of Ascending Aorta

### 4.1. In Vivo Evaluation of Elastic/Biomechanical Properties of Ascending Aorta

The biomechanical properties of the ascending aorta have been studied using various approaches. An intraoperative video-based method was proposed to assess the local biaxial strains of the ascending thoracic aorta in patients who were undergoing open-chest surgery. Repeated biaxial strain measurements were obtained at low- and high-pressure conditions with reliable precision. This method may be used to further study the biomechanical properties of the ascending thoracic aorta in various relevant populations of patients who are undergoing open-chest surgery. The method builds a foundation for clinically relevant biomechanical modeling and mechanobiological profiling [26].

The imaging-based monitoring of the biomechanical properties of the aortic wall may help to identify the patients with a borderline aortic dilatation that require pre-emptive surgery. The biomechanical parameters characterizing the distensibility and mechanical elasticity of the ascending aorta may be assessed using the data of the aortic deformations during the cardiac cycle in routine practice based on an electrocardiogram (ECG)-synchronized MRI. Serial cardiac magnetic resonance (CMR) measurements demonstrate that ascending aortic distensibility declined in BAV patients even faster than it did in a comparison group with connective tissue disorders [27]. A prospective assessment of the biomechanical and elastic aortic properties based on the data of echocardiography, CT, and CMR imaging is promising for risk stratification [28,29,30], but it should be integrated with other predictors.

### 4.2. Young’s Elastic Modulus

**Figure 1:**
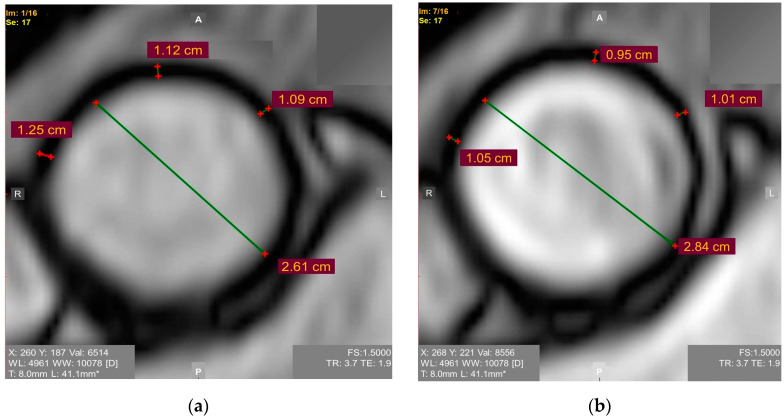
ECG-synchronized magnetic resonance aortography images used for Young’s modulus calculation in the ascending aorta: (**a**) Diastole. Mean diastolic aortic wall thickness = 1.15 mm; (**b**) Systole. Mean systolic aortic wall thickness = 1.05 mm.

Young’s elastic modulus is the ratio of the tensile stress to strain. Higher values of tensile strength and Young’s modulus are present in patients with an ascending aortic aneurysm [31], but studies focusing on Young’s modulus as a criterion for determining the timing of pre-emptive open aortic repair in patients with a borderline dilatation of the ascending thoracic aorta are almost lacking. We encourage researchers to consider the Young’s elastic modulus of the ascending aorta for the risk assessment of it because it may be easily calculated based on widely available ECG-synchronized MRI aortography as follows (1) (Figure 1) [28]:

E = ((d_diast_^2^ × (1 − 0.25) × ABP_pulse_)/(2 × h × ∆d_pulse_)) × 133.3 (1)

where

E—Young’s elastic modulus (Pa);
d_diast_—diastolic aortic diameter;
∆d_pulse_—an increase in aortic diameter during systole;
0.25—squared Poisson ratio for the aorta wall (Poisson ratio is known to be 0.5);
h—aorta wall thickness during diastole;
ABP_pulse_—pulse arterial blood pressure;
133.3—mmHg to Pa conversion factor.

A group of patients underwent ECG-synchronized MRI-based aortography for months before some of them developed an ascending aortic aneurysm. The values of the Young’s elastic modulus in all of the patients who later developed the aneurysms exceeded 0.67 mPa, though the study was limited by its retrospective nature and small sample size [28]. The involvement of the aorta wall thickness in the formula agrees with the report revealing the association between this parameter and the adverse ascending aortic events. It is expected that increased the aortic wall thickness may be the early warning sign requiring patient monitoring [32]. Considering that the biomechanical properties of the ascending aorta are anisotropic [33], it is essential to develop a well-defined standardized approach to assessing the Young’s elastic modulus.

### 4.3. Windkessel Function

The capacitance or the Windkessel function characterizes the aorta acting as an elastic buffering chamber behind the heart. The aorta stores about 50% of the left ventricular stroke volume during systole, and it releases the elastic forces during diastole. Energy loss is the measure of the Windkessel function. Recently, the biomechanics of normal, dilated, and dissecting ascending aortas were studied via destructive testing [34,35]. The energy loss under the biaxial tensile loading was shown to be independent of the strain rate, strain magnitude, and strain pre-load over a wide range of physiological values except at the low-strain regions. However, the energy loss was significantly correlated with the delamination strength. Energy loss may be considered a biomarker of aortic dissection risk in patients with ascending aortic aneurysms. [34,35].

### 4.4. Fluid Hemodynamics and Vortical Patterns in Proximal Aorta for Risk Assessment

One of the potential areas of growth in identifying the candidates for pre-emptive open aortic repair is the study of fluid hemodynamics and vortical patterns in the proximal aorta because the ascending thoracic aortic aneurysm progression may be partially flow-mediated [21]. The first attempts to understand hemodynamics in the aorta date as early as 500 years ago. Leonardo da Vinci depicted (~1512–13) multiple systolic flow vortices through the ascending aorta. Bissell et al. encoded 3D vector fields to map the aortic root blood flow using time-resolved magnetic resonance techniques and questioned the existence of secondary vortices which were postulated by Leonardo da Vinci in the normal aorta [36]. However, multiple vortices in the sinuses and proximal ascending aorta, which were postulated by Leonardo, still may be involved in aortic dilatation, hypertension, and valvular diseases [37].

New hemodynamic tools allow us to study the vorticity scores, the qualitative helicity, the quantitative vorticity, the local normalized helicity, the abnormally directed velocity volume, and the wall shear stress (WSS) surface in ascending aorta disease [38]. A WSS topological skeleton may reveal the promising indicators of local wall abnormalities [39]. According to a 4D flow CMR study, the helices and vortices in the thoracic aortic blood flow are present in aortic dilatations and aneurysms [39]. Aortic valve abnormalities cause aberrant 3D hemodynamics, and an altered WSS may potentially trigger an aortic dilatation and aortopathy [40,41].

A valuable tool was developed to study the hemodynamic alterations in the diseased aorta depending on the aortic shape and the aortic valve variations which were based on statistical shape modeling. The tool was designed to support the risk assessment in patients with thoracic ascending aortic disease over time. The authors created the ascending thoracic aortic aneurysm template, which may be modelled to see the differences that it produces in the flow patterns, blood pressure, and fluid shear forces when they are deformed. The authors performed the exquisite atlas-based analyses to understand the mechanistic link between the shape and the pathophysiology [42].

The hemodynamic patterns depend on the blood flow eccentricity at the aortic root regardless of the aortic valve phenotype [43]. The blood flow vortices produce the excessive mechanical stresses at certain aortic wall locations, which further become the foci of the dilatation and, eventually, they become the entry sites for acute aortic dissections in patients with aortic arch variants. The wall stress/strength relationships may be used for the risk assessment [44]. A turbulent blood flow may trigger signaling cascades in the aortic wall, and this is linked to an elevated eNOS expression at the concave wall in dilated aortas [45].

Regional hemodynamic and biomechanical characteristics depend on different rotational positions of the normal aortic root [46]. The velocity and therefore, the stresses over the aortic arch can be significantly reduced by a reversed flow and vortex formation as shown in both the normal and aortic arch aneurysm models [47].

Computational fluid dynamics enable us to evaluate the vorticity, the helicity index, a disturbed laminar flow, the flow streamline, the flow velocity, the WSS, and the recirculation regions in untreated and treated aortic diseases, and other fluid dynamic parameters [44,47,48,49].

## 5. Phenotype

The phenotypical “associates” of the silent thoracic aortic aneurysm may help in detecting this condition at an early stage. They include BAV, giant cell arteritis, having a family history of aortic aneurysms, positive thumb-palm sign [50], an intracranial aneurysm, an anomalous aortic arch, an aneurysmal abdominal aorta, and simple renal cysts [51,52]. The 15% prevalence of inguinal hernias in thoracic aortic disease exceeds that which is in a general population [53]. However, even if the cardiovascular surgeons are aware of these associations, other medical specialists sometimes lack this awareness, and so we encouraged the wider medical community to acquire this knowledge.

### 5.1. BAV

The BAV is one of the most significant phenotypical associates requiring attention. The TTE is the imaging technique of choice to diagnose a BAV. The measurement of the aortic root diameter should be made perpendicular to the axis of the proximal aorta, and it should be recorded from several slightly differently oriented long-axis views. The standard measurement is taken as the largest diameter from the right coronary sinus of Valsalva to the posterior (usually noncoronary) sinus. Most of the studies report aortic root diameter measurements at the end-diastole using the leading edge-to-leading edge technique [20]. The TTE is less helpful in examining the aortic root, the proximal ascending aorta, the mid-distal ascending aorta, and the arch. The CMR and CT provide better assessments of the aortic diameters based on multiplanar reconstructions [54]. A recent study supports the current guideline of the American Association of Thoracic Surgery, suggesting that no open aortic repair is needed in patients with BAV disease with an aortic root or ascending aorta diameter of 4.0–4.5 cm during an aortic valve replacement surgery. Indeed, the patients with an aortic valve replacement and a proximal thoracic aorta that is 4.0–4.5 cm have similar long-term survival rates, reoperation rates, and aorta growth rates when they are compared to the patients with the <4 cm proximal aortas. However, the authors still encourage the performance if long-term studies to make definitive recommendations and provide the adequate surveillance of this disease with cardiovascular imaging: CT angiogram, MRI, or echocardiography [55].

### 5.2. Aortic Arch Variants

Thoracic aortic disease is detected twice as often in individuals with anomalies of the aortic arch such as a bovine aortic arch, an isolated left vertebral artery, and an aberrant right subclavian artery. Atypical branching variants are potential anatomic markers predicting thoracic aortic disease [56]. A majority of patients (74%) with aneurysmal aortic arch branch vessels have an aneurysm that is located elsewhere in the body, most often in the ascending aorta (47%). A bovine aortic arch and an aortic arch branching variant are observed in 29% of the patients with the aneurysmal aortic arch branch vessels and 35% of the patients with an innominate aneurysm [57].

### 5.3. Thumb Palm Test

The “thumb palm test” is often positive (with the thumb crossing beyond the edge of the palm) in ascending aortic aneurysm patients. The sensitivity of the test is low (7.5%), but specificity of it is high (98.5%) [58].

### 5.4. Paradoxes

Interestingly, there is a paradoxical beneficial effect of diabetes on the thoracic aortic catastrophe risk. Diabetic patients have a slower rate of the aortic aneurysm growth, a lower aortic dissection rate, an older age at which the rupture occurs (>65 years), a lower mortality rate, a reduced hospital stay, an attenuated matrix metalloproteinases, and an increased aortic wall stress. The clinical and experimental studies demonstrate the ability of antidiabetic medications to inhibit the aortic aneurysm growth. Metformin, thiazolidinediones, and dipeptidyl peptidase-4 inhibitors may protect against an aneurysm and inhibit its growth [59]. Another paradoxical finding is that the diagnosis of an ascending aortic aneurysm is associated with a significantly reduced risk of systemic atherosclerosis [50]. However, the current recommendations do not suggest taking into consideration these comorbidities while weighing the risks of the adverse ascending thoracic aortic dilatation in the context of pre-emptive aortic repair.

## 6. Risk Associated with COVID-19 Infection

New risk factors may emerge due to the ongoing pandemic of the coronavirus disease 2019 (COVID-19) infection. Indeed, a severe acute respiratory syndrome coronavirus 2 infection (SARS-CoV-2) can deteriorate the biomechanical properties of the ascending aorta [59]. A single-centered, observational study of 38 adult patients with COVID-19 in Wuhan, China showed that over 50% of the patients had an increase in their ascending aorta diameter, which was positively associated with the C-reactive protein and creatine kinase myocardial band, and it was negatively associated with the lymphocyte count. An ascending aortic dilatation was associated with severe inflammation and a cardiac injury. Though an ascending aortic dilatation could potentially exist before the emergence of COVID-19, these findings are essential for identifying the high-risk patients with SARS-CoV-2 [60]. Children with SARS-CoV-2 and multisystem inflammatory syndrome have a decreased echocardiography-based ascending aortic strain and aortic distensibility values, which correlates with an attenuated flow-mediated dilatation [61].

## 7. Machine Learning

Deep learning techniques integrating the imaging data, the electronic health record data, and genetic sequences [62,63,64,65,66,67,68] are expected to contribute to breakthroughs in the aortic disease. They allow for increasing the sample size and improving the timeframe of the discoveries. Machine learning was shown to be capable of successfully diagnosing aortic aneurysms and predicting aortic complications [24]. A classic algorithm of machine learning that is called Random Forest does classification or regression by combining the voting results of multiple decision trees, and it improves prediction accuracy without significantly increasing the amount of computations that are needed. A Random Forest classifier that was trained on 1133 patients was able to predict the in-hospital rupture of the ascending aorta with a high degree of accuracy (AUROC of 0.752; sensitivity of 0.99) [14].

The study implementing a deep learning approach on aortic MRI data from the UK Biobank was able to measure the aortic diameter of over 30,000 individuals. This genome-wide association study identified the gene encoding supervillin (SVIL gene) , which is highly expressed in the vascular smooth muscle, as being significantly associated with both ascending and descending aortic dilatation [69].

The relationships between the aortic shape and the ascending aortic aneurysm rupture risk were studied via a machine learning approach using finite element analysis. The approach allowed for nearly 96% risk classification accuracy, though the authors used limited sets of imaging data to train the algorithm, whereas the material properties and the inhomogeneous thickness factors were not included [70].

**Figure 2:**
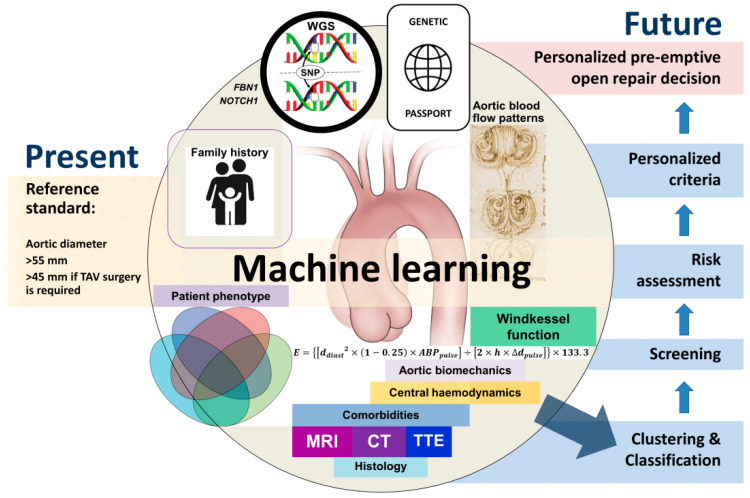
Proposed transdisciplinary approach to early risk assessment of ascending thoracic aortic aneurysm (MRI: magnetic resonance imaging, CT: computed tomography, TTE: transthoracic echocardiography, TAV: tricuspid aortic valve).

Machine learning may become a promising technique allowing for the personalization of the risk assessment in patients with ascending thoracic aortic dilations. However, we expect that there will be many more parameters which will be integrated into the machine learning algorithms to provide an appreciable discrimination power (Figure 2).

## 8. Comments

Though not all of the reviewed approaches are immediately applicable to the daily clinical practice, the calculation of Young’s elastic modulus and the assessment of central hemodynamics are fairly straightforward techniques for routine clinical practice. They may be readily used with the available equipment, requiring minimum additional training of the imaging specialists. In fact, the main idea of the approach that is presented here in this review, is the real necessity of the practical implementation of mechanical parameters to the routine clinical practice activity in diagnostic and surgical angiology, for example, in the first line, the use of the individually calculated Young’s modulus for the individual prognosis of patients with extensive aortic disease. The matter of the implementation of such an approach to everyday clinics can only be achieved through the development of attractive software that can deliver a three-dimensional distribution of the Young’s modulus values throughout the wall of aorta from the ECG-gated MRI-aortography. Currently, the design of such specialized packages is in progress.

Some of the discussed technologies require the sophisticated processing of the data, a complicated interpretation of it, and additional time and resources, whereas the majority of clinicians are not acquainted with these techniques. However, during the next decade, even the most sophisticated approaches for the management of patients with ascending aortic dilatation may be incorporated into the daily clinical practice in leading specialized centers. The practical implementation of transdisciplinary algorithms would require an integration of the advanced imaging modalities, a phenotypic assessment, genetics, machine learning, clustering, and neural network-based algorithms supporting the decision-making in selecting candidates for preemptive open aortic repair.

The main obstacle to implementing the transdisciplinary approach is the lack of coordination between the specialists from different disciplines, the fragmentation of the data, and the unawareness of the untrained clinicians. Data, which are acquired by using different techniques and produced by specialists whose professional paths do not overlap, should be integrated like fragments of a puzzle. The way to develop the transdisciplinary effort may be a creation of a cloud platform with different domains to provide an efficient clustering of the patients/candidates for surgery.

During the next decade, the authors of this paper also plan to develop and implement a formula for calculating the risk of surgery based on clinical, genetic, tomographic, and echocardiography data, taking into account the anatomic determinants and functional status of the aortic wall in the patients with an ascending aortic dilatation. This risk of the surgery will be weighed against the risk of the complications in marginally dilated ascending aortas and aortic roots. Such an approach would allow for us to make a balanced decision regarding the best personalized time for the open reconstruction of the ascending aorta and/or aortic root. The second task may be achieved through the use of a machine learning in a framework of the above-mentioned transdisciplinary approach.

It should be emphasized that the risk assessment of the complications in a moderately dilated ascending thoracic aorta/aortic root essentially differs from that in the descending thoracic aorta. The problem of the dissection of the ascending aorta is associated with a higher mortality rate and there being less time for decision-making than there are in the type B dissection. Moreover, the type B dissection may be treated less invasively, non-surgically or endovascular, in many world centers. Overall, the aneurysm of the descending aortas may be dynamically monitored for a longer time. It is believed that the size of it does not matter much in predicting acute type B aortic dissection [71]. The problem of risk prediction in ascending aortic disease seems more challenging than that in descending aortic disease. Moreover, even in embryogenesis, there are the essential differences, namely: the ascending aorta develops as a component of the primitive heart tube with the truncus arteriosus as the basis for the development of the ascending aorta; the arch of the aorta develops from multiple structures; the descending aorta arises from the dorsal aorta [72]. Therefore, we encourage a differentiated approach to the risk assessment of the complications in the ascending and descending thoracic aortas, which is consistent with the available literature.

In summary, a clinically relevant identification of the predictors is based on non-invasive or, sometimes, semi-invasive, imaging techniques such as MRI, CT, and ultrasound, and the current guidelines recommend the use of the aortic diameter-based assessment of the thoracic aortic aneurysm risk. However, thoracic aortic dilatation is a heterogeneous condition, and over 80% of ascending aorta dissections might occur at a size that is lower than the recommended threshold of 55 mm. Several alternative approaches have been proposed to predict the risk of dissection in patients with a borderline-dilated thoracic aorta, namely: measuring the alternative aortic dimensions (aortic length and aortic size indexes), assessing the biomechanical aortic properties (Young’s elastic modulus, compliance, distensibility, wall shear stress, and pulse wave velocity), the energy loss, genotyping and integrating all of the above with the patient’s phenotypes (hernias, BAV, aortic arch variants, thumb palm test, etc.). The standardized assessment of the mechanoelastic properties may be a promising, straightforward approach for an early risk assessment, but further research is required as the studies in this direction are nascent. New risk factors for thoracic aortic disease have emerged due to the ongoing coronavirus disease 2019 (COVID-19) infection pandemic. The epidemiological rates and patterns of ascending thoracic aortic dilations/aneurysms require continuous attention and systematic multi-center screening programs to identify the prevalence and diagnostic yields of ascending thoracic aortic disease. More sensitive indices are warranted to better discriminate between the aortopathy types to develop tools to select patients for the early pre-emptive surgery. While we do not argue for shifting the diameter threshold to the left, we do emphasize the need for more personalized solutions that integrate the imaging data with the patient’s genotypes and phenotypes in this heterogeneous pathology. Taking into account the sixth techno-economic paradigm, we conclude that interdisciplinary methods are not enough to further advance the state of the knowledge regarding the practice-oriented assessment of the ascending aortic aneurysm risk. Instead, it requires the use of transdisciplinarity methods and the emergence of new scientific disciplines, allowing for researchers to build the effective virtual cognitive centers. Meanwhile, a purely aortic diameter-based assessment of the thoracic aortic aneurysm risk will remain the standard of care.

## Data Availability

To find the relevant literature data, the electronic databases including PubMed/Medline (1966–2022), Web of Science (1975–2022), Scopus (1975–2022), and Russian Science Citation Index (RSCI) (1994–2022) were searched.
